# Coherence for nutrition: insights from nutrition-relevant policies and programmes in Burkina Faso and Nigeria

**DOI:** 10.1093/heapol/czab108

**Published:** 2021-09-21

**Authors:** Lucy Billings, Rebecca Pradeilles, Stuart Gillespie, Anna Vanderkooy, Dieynab Diatta, Mariama Toure, Ampa Dogui Diatta, Roos Verstraeten

**Affiliations:** Poverty Health and Nutrition Division, International Food Policy Research Institute, 1201 Eye Street, NW, Washington, DC 20005 USA; School of Sport, Exercise and Health Sciences, Loughborough University, Clyde Williams Building, Epinal Way, Loughborough LE11 3TU UK; Poverty Health and Nutrition Division, International Food Policy Research Institute, 1201 Eye Street, NW, Washington, DC 20005 USA; Independent consultant; Poverty Health and Nutrition Division, International Food Policy Research Institute, 1201 Eye Street, NW, Washington, DC 20005 USA; Poverty Health and Nutrition Division, International Food Policy Research Institute, 1201 Eye Street, NW, Washington, DC 20005 USA; Poverty Health and Nutrition Division, International Food Policy Research Institute, 1201 Eye Street, NW, Washington, DC 20005 USA; Poverty Health and Nutrition Division, International Food Policy Research Institute, 1201 Eye Street, NW, Washington, DC 20005 USA

**Keywords:** nutrition, policy, programme, coherence, Nigeria, Burkina Faso

## Abstract

There is consensus that policy coherence is necessary for implementing effective and sustainable approaches to tackle malnutrition. We look at whether policies and programmes provide a coherent pathway to address nutrition priorities and if programmes are designed to deliver interventions aligned to the nutrition policy agenda in Nigeria and Burkina Faso. A systematic desk review was performed on nutrition-relevant policy and programme documents, obtained through grey literature searches and expert recommendations. We developed a framework with an impact pathway structure that includes five process steps, which was used to guide coding, data reduction and synthesis and structure the analysis. We assessed internal coherence along process steps within a given document and external coherence across process steps for explicitly linked policy/programme pairs. The majority of policies and programmes had partial internal coherence for both countries. The identification of relevant nutrition interventions to address challenges and reach objectives was the strongest connection within policies (16 out of 45 had complete coherence), while among programmes, the strongest connection was coverage indicators that measure interventions (9 out of 21 had complete coherence). Eight programmes explicitly referenced at least one nutrition-relevant policy, with a total of 16 linked policy/programme pairs (13 pairs for Burkina Faso and 3 for Nigeria) across health, nutrition, agriculture and social focus areas. However, none of the linked pairs were assessed to have complete external coherence, suggesting that priorities at the policy level are not fully realized nor translated at the programme level. This study offers a new approach for the assessment of policy and programme coherence and specifically examines policy and programme linkages. We conclude that improved leadership on country priority setting and better alignment for nutrition within and across sectors is needed to enhance the effectiveness of nutrition investments.

Key messages

- The results of this coherence analysis shed insights on opportunities for greater alignment on the content within and across policies and programmes.
- Priorities for action are leadership and coordination on nutrition, which will help to ensure that national nutrition priorities, as elaborated in policy documents, are reflected at the programme level.
- Integration of nutrition data and evidence for each step along an impact pathway will ensure consistency and coherence within and across nutrition-relevant policies and programmes.
- Improved coherence within and across documents increases the likelihood that policies and programmes reach their intended outcomes, such as improved nutrition.

## Introduction

There is strong evidence regarding the drivers of, and solutions to, key global nutrition challenges in children under five (U5) and in women of reproductive age (WRA) (e.g. stunting, wasting, micronutrient deficiencies and overweight/obesity), drawing on robust empirical research and experience (Arenz *et al.*, 2004; Checkley *et al.*, 2008; Imdad and Bhutta, 2011; Kramer and Kakuma, 2012; Bhutta *et al.*, 2013; Horta and Victora, 2013; Malik *et al.*, 2013; Te Morenga *et al*., 2013; Peña-Rosas *et al.*, 2015; Low *et al*., 2016; Keats *et al.*, 2021). More recently, double-duty actions have been proposed to simultaneously tackle multiple forms of malnutrition (Hawkes *et al.*, 2020). With this wealth of evidence, we would expect widespread implementation of lifesaving and life-improving interventions and rapid improvement in nutrition outcomes. Yet, in many low- and middle-income countries (LMICs), progress has been inconsistent and varies both within and between countries (World Health Organization, 2013; Development Initiatives, 2020; Heidkamp *et al.*, 2021). In the African region, only 8 out of 54 countries are ‘on course’ to meet the global nutrition targets for U5 stunting, 12 for U5 wasting, 20 for U5 overweight and 20 for exclusive breastfeeding (Development Initiatives, 2020). No country in the region is on track to meet the targets for anaemia among WRA, low birthweight (LBW) and male/female diabetes and obesity. The unequal progress within and across countries has been attributed to several factors, including political instability; a lack of resources; the inadequate implementation of policies, programmes and interventions; the lack of coherence and coordination across different sectors (Development Initiatives, 2020) and policy process constraints (Pelletier *et al.*, 2012).

In order to maximize progress in nutrition outcomes, the policy literature places particular emphasis on the importance of an ‘enabling environment’, defined as the political and policy processes that build and sustain momentum for the effective implementation of actions that reduce all forms of malnutrition (Gillespie *et al.*, 2013; World Health Organization, 2013; Gillespie *et al.*, 2015). Given the complex landscape of nutrition in LMICs, a unified nutrition agenda is needed at country level to guide, monitor and evaluate strategic actions across sectors and at different levels of government according to identified needs and priorities (Gillespie *et al.*, 2013; 2019; Hawkes, 2016). This requires a high degree of policy coherence, which entails ‘consistency, comprehensiveness and harmonious-compatible outcomes across policy areas and sectors without compromising the integrity of policymakers’ goals’ (Dubé *et al.*, 2014) in addition to consistency within the different elements of a given policy from goals to actions, implementation and outcomes (Huttunen *et al.*, 2014). Policy coherence analysis has been identified as critical to achieve the sustainable development goals (SDG Target 17.14) (United Nations, 2015) and promises to be an important tool to improve the efficiency and effectiveness of food policy and practice (Parsons and Hawkes, 2019). Heidkamp *et al*. (2021) demonstrated that coherence in planning and action within and across sectors relevant for nutrition is a key enabler of significant progress in reducing undernutrition. While there is ample research on cross-sector policy coherence for nutrition, an important and understudied element of coherence is the degree of alignment between policies and programmes. In this study, we applied a novel approach to empirically assess coherence for nutrition across policies and programmes relevant for nutrition (i.e. those that address immediate or underlying determinants of nutrition).

The West African subregion faces a particular challenge with the double burden of malnutrition, characterized by a high prevalence of forms of undernutrition, among children and WRA, and a rapid rise in overweight/obesity and nutrition-related non-communicable diseases (NR-NCDs) among the adult population (Verstraeten and Diop, 2018; Development Initiatives, 2020; Popkin *et al.*, 2020). The two countries selected for this study, Nigeria and Burkina Faso, both exhibit a high prevalence of malnutrition. The prevalence of U5 stunting is 43.6% in Nigeria and 21.1% in Burkina Faso, while the prevalence of U5 wasting is 10.8% in Nigeria and 8.6% in Burkina Faso (Development Initiatives, 2021a,b). Moreover, large disparities exist in both countries, with certain areas such as the Sahel and the northern regions of Nigeria particularly affected by these forms of undernutrition (Institut National de la Statistique et de la Démographie (INSD), 2012; United Nations Children’s Fund (UNICEF), 2012; National Population Commission, 2019). Among WRA, the prevalence of anaemia is 49.8% in Nigeria and 49.6% in Burkina Faso, while 13.1% of women in Nigeria and 8.1% of women in Burkina Faso are obese (Development Initiatives, 2021a,b). Both countries have invested in institutional transformations bringing people together into a shared space for action, ensuring a coherent policy and legal framework, aligning actions around common results, and financing tracking and resource mobilization for nutrition to address these outstanding nutrition challenges. These transformations increased steadily in Burkina Faso over the past years but are much more recent for Nigeria (Scaling Up Nutrition, 2019a,b). In this study, we seek to determine if (1) nutrition-relevant policy and programme documents are coherent in their pathway to address key nutrition priorities and (2) whether policies and programmes are aligned to effectively address these priorities, meaning the delivery of programmes to operationalize nutrition-relevant policies. In this study, we consider a policy or programme nutrition-relevant if it has an objective, budget or indicator for nutrition.

## Methods

We performed two parallel country case studies for Nigeria and Burkina Faso. We conducted a comprehensive systematic desk review of policy and programme documents to examine coherence within and across nutrition-relevant policies and programmes, using an a priori defined protocol.

### Analytical framework

**Figure 1.**
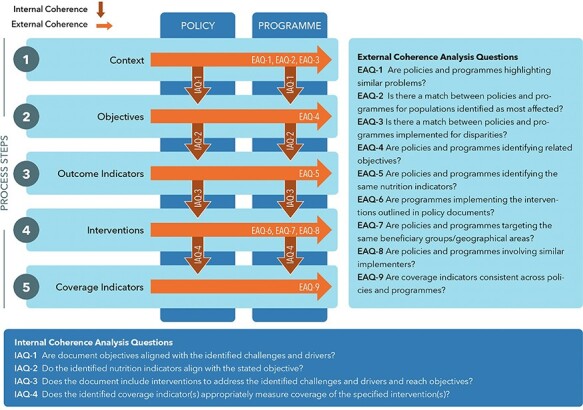
Coherence matrix of nutrition relevant policies and programmes

Policy coherence analysis is a relatively new methodological approach, with no standardized and validated method to assess coherence (Nilsson *et al.*, 2012; Parsons and Hawkes, 2019). Studies across various fields have used different definitions of coherence and analytical approaches (e.g. environment and climate; Nilsson *et al.*, 2012; Huttunen *et al.*, 2014); food security and nutrition (Fox *et al.*, 2015; Thow *et al.*, 2018); trade and nutrition (Baker *et al.*, 2019); and agriculture and climate (Harahap *et al.*, 2017). All of these approaches assess the interplay (1) across different layers of a policy from policy goals to actions, implementation and outcomes and (2) between policies in various sectors (Nilsson *et al.*, 2012; Huttunen *et al.*, 2014; Parsons and Hawkes, 2019). None of these approaches, however, seeks to assess the policy–programme nexus. Guided by these previous studies, we defined an analysis framework to assess coherence for nutrition within and across policies and programmes. The framework includes five process steps following an impact pathway structure: (1) identification of key nutrition challenges in a landscape or ‘context’ analysis, (2) specification of priority nutrition ‘objectives’ to address the identified challenges, (3) identification of ‘outcome indicators’ (nutrition status or nutrition drivers) to measure progress towards the stated objectives, (4) identification of relevant nutrition ‘interventions’ to address nutrition challenges and reach objectives and (5) identification of relevant ‘indicators to measure intervention coverage’ and track service delivery (Figure 1). Similar to Huttunen *et al*. (2014) and Nilsson *et al*. (2012), we use this analytical framework (Figure 1) to guide the data coding, reduction, synthesis as well as to assess coherence for nutrition. We define ‘internal coherence’ as the alignment of context, objectives, interventions and indicators within a given policy or programme and ‘external coherence’ as whether this alignment is compatible across policies and programmes.

### Identification of policy and programme documents

#### Inclusion and exclusion criteria

Nutrition-relevant policies and programmes for Burkina Faso and Nigeria that were implemented, in use, or in an advanced drafting stage as of December 2018 (for policies) and August 2019 (for programmes) were included in this review. We included ‘policies’ (government policies, strategies or action plans), ‘programmes’ (operational/implementation plans, strategy programmes or action plan, and interventions that serve as a policy operationalization or delivery tool), and ‘policy/programme documents’ (combination of policies and programmes in one document). Policy and/or programme documents were considered nutrition-relevant and eligible for inclusion if they (1) were either nutrition-specific (i.e. addressing immediate determinants of nutrition) or nutrition-sensitive (i.e. addressing underlying determinants of nutrition); (2) had a nutrition-sensitive or nutrition-specific objective aiming to address at least one nutrition outcome; or a budget for a nutrition activity/intervention (documents with an overall budget which incorporated nutrition were included); and/or a nutrition indicator (related to nutritional outcomes, behaviour; e.g. dietary diversity), or coverage of nutrition interventions); and (3) were implemented at national (Burkina Faso) level or drafted at federal level even if only implemented at sub-national level (Nigeria). Policies were excluded if they were not endorsed at national (Burkina Faso) or federal (Nigeria) level or when they were developed by non-governmental organizations (NGOs) and international NGOs (INGOs) unless the government had adopted these policies for country implementation.

#### Search strategy

The following complementary search approaches were used to identify policies and programmes at country level: (1) targeted website search including government ministries, development agencies, NGOs and INGOs; (2) Google search with defined search terms (first 100 hits were screened for inclusion) and (3) consultation with content experts representing government offices, NGOs and INGOs in-country via email/phone. The key search terms that were used included Nigeria or Burkina Faso and policy or programme terms (both in English and in French) combined with nutrition. The detailed search strategy is presented in Supplementary Material 1. Searches were completed in December 2018 (for policies) and August 2019 (for programmes) by AV and MT, respectively; any documents released or updated after these dates were not included in this review.

#### Screening process

All retrieved documents from the various sources were entered in Excel, and duplicates were removed before screening. All documents were screened against the established inclusion criteria by AV and RV (policies) and MT, AD, RP and LB (programmes); screening results were logged in Excel. To ensure consistency in the application of the inclusion criteria, an independent reviewer screened all excluded documents (RV, LB or RP). Spot checks revealed strong congruence across reviewers, with any discrepancies resolved through discussion.

### Data coding and synthesis

The coherence analysis framework (Figure 1) served to guide the coding, reduction and synthesis of the qualitative data. We used systematic document analysis (Bowen, 2009) applying a framework method approach (Srivastava and Thomson, 2009; Gale *et al.*, 2013) to collate information from the documents for the assessment of coherence.

#### Data coding and extraction

Descriptive characteristics were extracted for each retained document for title, focus area [including sectors (e.g. agriculture, health, education) and multi-sector areas (e.g. nutrition, WASH (water, Sanitation, and Hygiene), crosscutting)], organization, type of document, and the programme/policy start and end date. The process steps in the analytical framework (context, objectives, indicators and interventions) provided the structure for a pre-defined coding tree (i.e. deductive thematic content analysis) (Supplementary Material 2) to code retained documents. The coding tree was piloted and revised following discussion and agreement by the research team. Data coding was undertaken in NVivo 12 (12.5.0) for the included policy (AV, AD and RV), programme (RP) and policy/programme documents (RP). The coded data were extracted into a standardized data extraction spreadsheet that included context (forms of malnutrition, drivers, consequences, populations affected, disparities and whether the situation analysis was evidence-based or not), objectives (general and nutrition objectives), nutrition indicators, intervention [type and content, population coverage, implementers, financing (funding and costing), coordination mechanisms] and coverage indicators. The data extraction form was piloted by two researchers, modified following discussion and then finalized (Supplementary Material 2).

#### Data reduction and synthesis

The following steps were taken for the assessment of coherence: (1) data reduction: to reduce the data while retaining the original meaning of the text, we summarized the extracted data into a narrative summary matrix for each process step for all retained policy and programme documents; (2) data synthesis: we assessed coherence for nutrition using analysis questions mapped to the process steps. We included four internal analysis questions (IAQ 1–4) and nine external analysis questions (EAQ 1–9), for assessment of internal and external coherence, respectively (Figure 1). Coherence was assessed for each analysis question from the corresponding narrative summary matrix according to three categories of coherence: none, partial or complete. The assessment categories were used to provide a quantitative summary of findings. The descriptive characteristics are visualized for each document by analysis question using the categorical assessment. The in-depth synthesis included a qualitative analysis of the coherence assessment to identify reasons contributing to lower or stronger coherence.

## Results

### Characteristics of included documents

**Figure 2.**
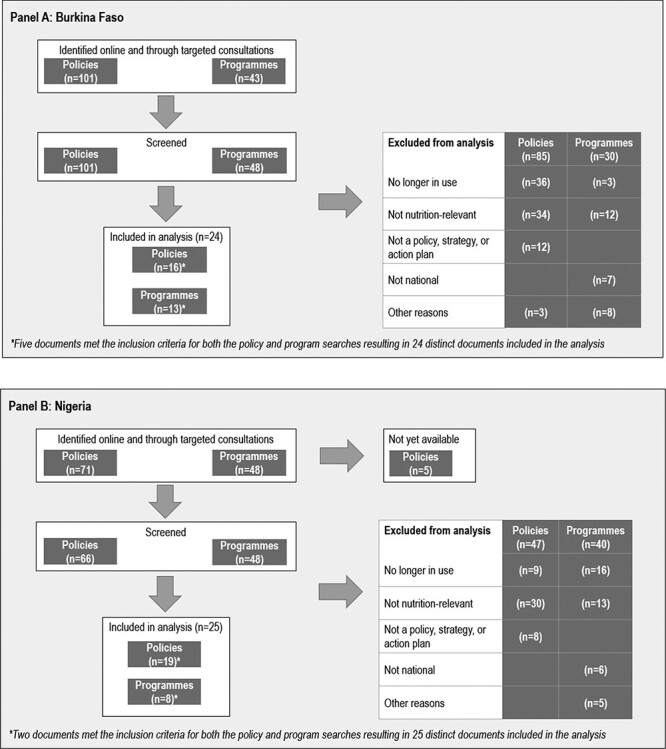
Study flow charts for (A) Burkina Faso and (B) Nigeria

For Burkina Faso, we retained 24 documents which included 11 policies, eight programmes and five policy/programme documents for analysis (Figure 2, panel A). From the 25 documents retained for Nigeria, 17 were policies, 6 were programmes and 2 were policy/programme cross-over documents (Figure 2, panel B). Policies and programmes were found in eight different focus areas across both countries, including agriculture, education, environment, health, nutrition, social, WASH and crosscutting. Duration of policies and programmes ranged between 3 and 12 years. Most policies and programmes had start dates within the past five years, with a few that had start dates as far back as 2006. End dates mostly ranged from 2020 to 2025, with a few cases where no end date was specified (Table 1). The included policies and programmes were numbered for Burkina Faso (BF1-BF24) and for Nigeria (N1-N25); we use these numbers to reference the documents throughout the manuscript. Policy/programme cross-over documents that were included in both the policy and programme analyses of internal coherence are denoted with an asterisk (BF3*, BF6*, BF9*, BF12*, BF15* and N16*, N17*).

**Table 1.** Descriptive details of policy and programme documents included for Burkina Faso and Nigeria

Burkina Faso										
	Policies					Programmes				
Focus area	#	Name	Acronym	Start	End	#	Name	Acronym	Start	End
Agriculture	BF1	Politique Nationale de Sécurité Alimentaire et Nutritionnelle (‘Politique Nationale de Secruite Alimentarie et Nutritionelle’, 2013)	PNSAN	2013	2025	BF17	Sécurité alimentaire et renforcement de la résilience (German Agency for International Cooperation, 2018)	–	2014	2022
	BF2	Politique Sectorielle Production Agro-sylvo-pastorale (Ministère de l’agriculture et des aménagéments hydrauliques, 2017)	PS-PASP	2017	2026	BF18	Programme d’appui à la sécurité alimentaire et nutritionnelle, a l’agriculture durable et à la résilience au Burkina Faso	PASANAD	2017	2021
	BF3^a^	Priorités Résiliences Pays (National Government of Burkina Faso, 2015)						PRP-AGIR	2016	2020
	BF4	Stratégie de Développement Rural (‘Stratégie de Développement Rural (SDR à l’horizon 2025)’, 2016)	SDR-2025	2016	2025					
Education						BF19	Politique sectorielle de l’éducation (National Government of Burkina Faso, 2013)	PSE	2014	2023
						BF20	Programme de développement stratégique de l’éducation de base (National Government of Burkina Faso, 2012)	PDSEB	2012	2021
Environment	BF5	Plan National d’Adaptation aux changements climatiques (Ministère de l’Environnement et des Ressources Halieutiques, 2015)	PNA	2015						
Health	BF6^a^	Plan National de Développement Sanitaire (Burkina Faso Ministère de la Santé, 2011)						PNDS	2011	2020
	BF7	Plan stratégique de santé des personnes âgées (Burkina Faso Ministère de la Santé, no date)	PSSPA	2016	2020	BF21	Health services reinforcement project (The World Bank, 2018b)	-	2018	2023
	BF8	Plan stratégique intégré de la santé reproductive, maternelle, néonatale, infantile, des adolescents, des jeunes et de la personne âgée (Burkina Faso Ministère de la Santé, 2017)	SRMNIA-PA	2017	2020	BF22	Plan de passage à l’échelle de la promotion des pratiques optimales d’alimentation du nourrisson et du jeune enfant au Burkina Faso (Burkina Faso Ministère de la Santé, 2014)	-	2013	2025
	BF9^a^	Plan stratégique intégré de lutte contre les maladies non transmissibles (Burkina Faso Ministère de la Santé, 2016a)						PSLMNT	2016	2020
	BF10	Politique sectorielle Santé	PSS	2017	2026	BF23	Programme Indicatif National (Union européenne and Burkina Faso, 2014)	PIN	2014	2020
Crosscutting	BF11	Politique sectorielle de la Recherche et de l’innovation	PSRI	2017	2026					
Nutrition	BF12^a^	Plan Stratégique Multisectoriel de Nutrition						PSMN	2017	2020
	BF13	Politique Nationale de Nutrition (Burkina Faso Ministère de la Santé, 2016b)	PNN	2016		BF24	Draft Burkina Faso country strategic plan (World Food Programme, 2018)	CSP	2019	2023
	BF14	Stratégie nationale de plaidoyer, mobilization sociale, et communication pour le changement social et de comportement en faveur de la nutrition au Burkina Faso (UN Network for SUN, 2017)	SNNBF	2017	2021					
Social	BF15^a^	Plan National de Développement Economique et Social (National Government of Burkina Faso, 2016)						PNDES	2016	2020
	BF16	Stratégie Nationale de Développement Intégré de la Petite Enfance	SNDIPE	2007						
**Nigeria**										
Agriculture	N1	Agricultural Sector Food Security and Nutrition Strategy (The Federal Republic of Nigeria, 2017)	ASFSNS	2016	2025	N20	Livelihood Improvement Family Enterprises Project in the Niger Delta of Nigeria (IFAD and Federal Government of Nigeria, 2017)	LIFE-ND	2016	2021
	N2	Agriculture Promotion Policy (Nigeria Federal Ministry of Agriculture and Rural Development, 2016)	APP	2016	2020	N21	Value Chain Development Program (IFAD, 2012)	VCDP	2012	2021
	N3	National Agricultural Investment Plan (Development, 2010)	NAIP	2011	2014 (in use)					
Education	N4	National School Health Policy (Nigeria Federal Ministry of Education, 2006)	NSHP	2006		N22	Nigeria Home Grown School Feeding Strategic Plan (Federal Governmet of Nigeria, 2016)	HGSF	2016	2020
	N5	Science, Technology, and Innovation Policy (Government of the Federal Republic of Nigeria, 2011)	STIP	2011						
Environment	N6	National Forest Policy	NFP	2006						
Health	N7	Integrated Maternal, Newborn and Child Health (IMNCH) strategy	IMNCHS	2007	2015 (in use)	N23	Nigeria States Health Investment Project	NSHIP	2013	2020
	N8	National Child Health Policy (draft version)	NCHP	2017						
	N9	National Health Policy (Nigeria Federal Ministry of Health, 2016)	NHP	2016						
	N10	National Health Promotion Policy (Nigeria Federal Ministry of Health, 2006)	NHPP	2006						
	N11	National Strategic Plan of Action on Prevention and Control of Non-Communicable Diseases (Health, 2015)	NSPANCD	2016	2020					
	N12	Second National Strategic Health Development Plan (Federal Government of Nigeria, 2018)	NSHDP II	2018	2022					
	N13	Task-shifting and task-sharing policy for essential health care services in Nigeria (Nigeria Federal Ministry of Health, 2014b)	TSTS	2014						
Nutrition	N14	National Policy on Food and Nutrition (Nigeria Ministry of Budget and National Planning, 2016)	NPFN	2016	2025	N24	Partnership for Improving Nigeria Nutrition System	PINNS	2019	2021
	N15	National Policy on Infant and Young Child Feeding in Nigeria (Nigeria Federal Ministry of Health, 2010)	NPIYCF	2010		N25	Accelerating Nutrition Results in Nigeria project (The World Bank, 2018a)	ANRiN	2018	2023
	N16^a^	National Social and Behavioural Change Communication (SBCC) Strategy for Infant and Young Child Feeding (IYCF) in Nigeria 2016–2020						SBCC	2016	2020
	N17^a^	National Strategic Plan of Action for Nutrition (Nigeria Federal Ministry of Health, 2014a)						NSPAN	2014	2019
Social	N18	National Social Protection Policy	NSPP	2017						
WASH	N19	Partnership for Expanded Water Supply, Sanitation & Hygiene Strategy (Nigeria Federal Ministry of Water Resources, 2016)	PEWASH	2016	2030					

a Cross-over documents that met the inclusion criteria in both the policy and programme searches.

### Internal coherence

#### Descriptive analysis of internal coherence

**Figure 3.**
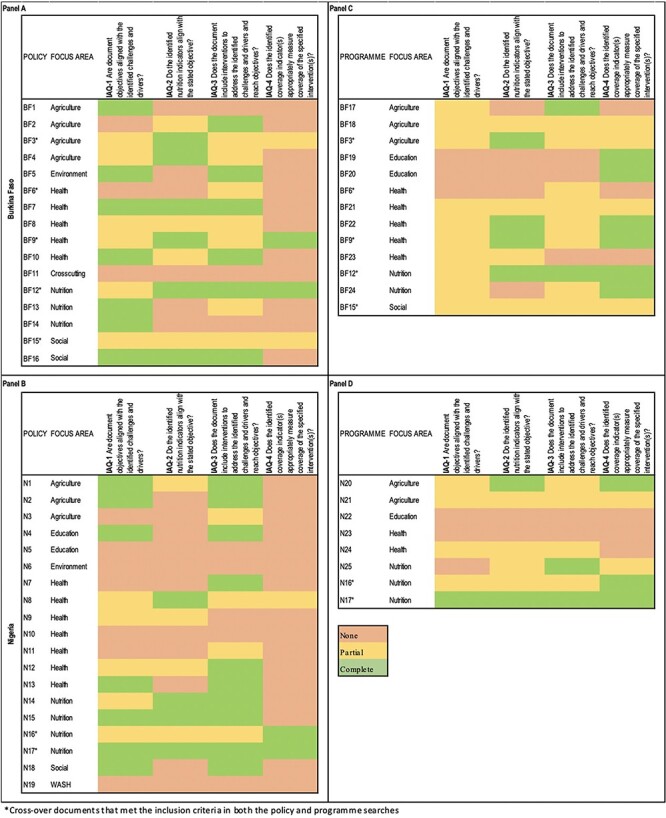
Internal coherence of policy and programme documents in Burkina Faso and Nigeria

The majority of policy and programme documents in both countries had partial internal coherence. The level of coherence varied for each individual document and by analysis question (Figure 3).

Two Burkina Faso policy documents (BF7 and BF16) from the ‘health’ and ‘social’ focus areas were assessed to have complete coherence for three of the four analysis questions. In Nigeria, one policy document (N15) with a ‘nutrition’ focus was assessed to have complete coherence for three of the four analysis questions. Four policy documents (N5, N6, N10 and N19), representing the ‘education’, ‘environment’, ‘health’ and ‘WASH’ focus areas, lacked coherence for nutrition across all analysis questions (Figure 3B).

Five programme documents had either one (BF17, BF19, BF20 and BF24) or two (BF22) analysis questions with complete coherence in Burkina Faso (Figure 3C). Two Nigeria programmes had either one (N25) or two (N20) analysis questions with complete coherence. Two programme documents (N22 and N23) from the ‘education’ and ‘health’ focus areas lacked coherence for all process steps (Figure 3D).

For policy/programme cross-over documents in Burkina Faso, one document in the focus area of *nutrition* had complete coherence for three of the four analysis questions (BF12*). Two documents were assessed to have complete coherence for one (BF3*) or two (BF9*) analysis questions. In Nigeria, both cross-over documents had a ‘nutrition’ focus, and of these one (N17*) had complete coherence for all four analysis questions while the other (N16*) had complete coherence for one analysis question and partial coherence for the other three.

#### Narrative synthesis of internal coherence

Among policies, the strongest connection was found for IAQ-3 (alignment between interventions and context), where 16 policies (45%) were assessed to have complete coherence. The weakest connection was found for IAQ-4 (alignment between coverage indicators and interventions), where only four policies (11%) were assessed to have complete coherence. However, among programmes, IAQ-4 had the highest level of coherence, with nine programmes (43%) assessed to have complete coherence. In both Burkina Faso and Nigeria, documents in the nutrition and health focus areas score better on internal coherence for nutrition compared to other focus areas. Complete lack of coherence for nutrition was more prevalent in policies and programmes in Nigeria (education, environment, health and WASH) when compared to Burkina Faso (cross-cutting).

In-depth analysis of the alignment between the objectives and the context (IAQ-1) for these policies and programmes revealed instances of a missing situation analysis, meaning challenges/forms of malnutrition were not described. In other instances, there was a lack of clarity in the objective (i.e. too broad without explicit links to nutrition) or absence of any nutrition objective, making it impossible to determine alignment between the objectives and the context (IAQ-1) for these documents (e.g. N5, N6, N22, N23, BF6*, BF11, BF20). We also found documents with both a situation analysis and objectives, but these were not aligned (e.g. BF12*, BF17). For IAQ-2 (alignment between objective and nutrition indicators), we found cases where the nutrition indicators were either missing completely or only mapped to some of the stated objectives but not all (e.g. N2, N18, BF1, BF5, BF6*). Other common types of misalignments included (1) inconsistency in target population across the document situation analysis, objectives and nutrition indicators (e.g. N12 in which policy challenges/drivers and objectives cover a wide range of population groups such as U5, WRA, elderly, adolescents and vulnerable groups, but nutrition indicators only cover U5 and WRA); and (2) inconsistency between the identified challenges, objectives and indicators (e.g. BF12* highlights LBW as a challenge and includes broad objectives that could encompass LBW but fails to include any indicators on LBW). Summary examples from the internal coherence analysis of policy and programme documents are presented in Table 2 by analysis question and assessment category.

**Table 2.** Summary examples from policy and programme documents by assessment category—internal coherence

	Level of coherence		
Analysis questions	None	Partial	Complete
IAQ-1. Do the objectives align with the identified challenges and drivers?	The general objective is ‘to deliver a government-led, cost-effective school feeding programme that will establish a safety net for the poor and eradicate malnutrition in school age children’. Nutrition challenges and drivers are not described in this document. There is, therefore, a lack of coherence between objectives and context. —Nigeria programme (N22)	The programme aims ‘to increase the quality and utilisation of health services with a particular focus on maternal, child and adolescent health, nutrition and disease surveillance’ through four main components; one of which was nutrition-related (i.e. strengthening delivery of Reproductive, Maternal, Newborn, Child and Adolescent Health and Nutrition (RMNCAH + N)). Within the integrated RMNCHA + N package, a set of nutrition-specific interventions would be scaled up to reduce stunting. This document specifically aimed at reducing stunting, excluding the other forms of malnutrition mentioned in the situation analysis, such as wasting in children and maternal malnutrition. Given that the document focused on improving the nutrition services, it is implied that by enhancing these, this would also benefit other forms of malnutrition and drivers; however, it was not clearly specified. Overall, the objectives partially aligned with the identified challenges and drivers. —Burkina Faso programme (BF21)	The programme aims ‘to improve the nutritional status throughout the lifecycle of Nigerian people, with a particular focus on vulnerable groups including WRA and children U5’. There are six priority areas of focus: maternal nutrition; IYCF; management of SAM under five; micronutrient deficiency control; diet-related NCDs; nutrition information system. Forms of malnutrition/WHA targets described in the context included stunting, wasting, low birthweight/IUGR, anaemia in women, undernutrition, micronutrient deficiencies, overweight and obesity. Key drivers included suboptimal IYCF, education, poverty, poor WASH practices. The objectives are in complete alignment with the identified challenges and drivers presented in the document’s situation analysis. **—**Nigeria policy/programme (N17*)
IAQ-2. Do the identified nutrition indicators align with the stated objective?	There are no nutrition indicators included in the document. Therefore, there is no alignment between nutrition indicators and the stated objective ‘to increase the delivery and use of high impact maternal and child health interventions and improve quality of care available to the people in Nasarawa and Ondo and all the States in the North East’. —Nigeria programme (N23)	The programme objective is ‘to increase utilization of quality, cost-effective nutrition services for pregnant and lactating women, adolescent girls and children under five years of age’ focused on infant and young child feeding (IYCF) behaviour change, including breastfeeding and complementary feeding and the related provision of micronutrient powders to children under 2 years. Delivery of services to treat severe acute malnutrition (SAM) in children 6–59 months is a secondary objective. A number of indicators are included on delivery of nutrition services. There are indicators for exclusive breastfeeding and behaviour change communication, but there are no indicators for continued breastfeeding or complimentary feeding, important components of IYCF practices. There is no mention of indicators on wasting or weight for age to measure changes in SAM. —Nigeria programme (N25)	The nutrition indicators identified are in full alignment with the overall objective to tackle diet related non-communicable diseases and to promote healthier diets, which are a driver of overnutrition. **—**Burkina Faso policy/programme (N9)
IAQ-3. Does the document include interventions to address the identified challenges and drivers?	The document identifies acute malnutrition among children under 5 and food insecurity and key nutrition challenges. However, no interventions are described in the document resulting in a missing link between nutrition challenges or drivers and interventions. —Burkina Faso programme (BF23)	Agricultural market development and smallholder productivity enhancement are programme interventions that aim to address food insecurity, which is identified as a driver of malnutrition. However, the document only generally identifies malnutrition as a challenge, without specifying forms of malnutrition. The alignment between interventions and objectives was assessed as partial since there is a clear link for food security but not sufficient specificity on how the interventions will address malnutrition. —Nigeria programme (N21)	A number of interventions are mentioned in this document (e.g. improving IYCF practices; iron and folic acid supplementation in pregnant women, children U5 and school aged children; vitamin A supplementation in 6- to 59-month-old children, post-partum mothers and school-aged children; deworming in children aged 12–59 months; nutrition education around food safety). All interventions would contribute to addressing the forms of malnutrition and drivers stated in the document’s situation analysis. —Burkina Faso policy/programme (BF12*)
IAQ-4. Does the identified coverage indicator(s) appropriately measure coverage of the specified intervention(s)?	No indicators for measuring intervention coverage are included in the document. Therefore, the link between coverage indicators and interventions is assessed as missing. —Burkina Faso programme (BF17)	Coverage indicators are included in the document for some of the specified interventions (i.e. community health workers receiving training on community-level IYCF and IMPC; people who have received essential health, nutrition and population services; women and children who have received basic nutrition services). However, no coverage indicators are listed to measure interventions to improve IYCF practices and micronutrient supplementation. —Burkina Faso programme (BF21)	The document includes indicators to appropriately measure coverage of the programme interventions that focus on behaviour change communication to improve infant and young child feeding practices at individual, community, and state/federal levels. —Nigeria programme (N16*)

### External coherence

#### Descriptive analysis of external coherence

**Figure 4.**
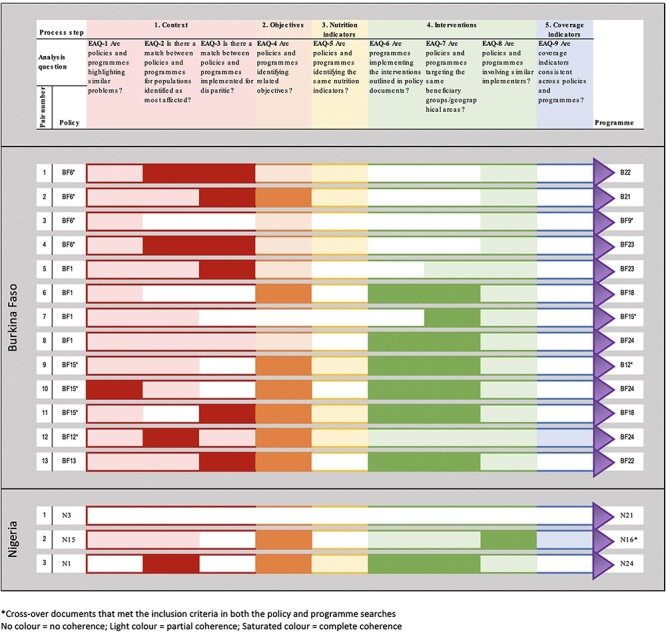
External coherence of matched policy and programme pairs for Burkina Faso and Nigeria

There was a striking difference in the number of programmes that explicitly referenced policies between the two case study countries. Only three policy/programme pairs were retained for the full external coherence analysis for Nigeria, each referencing a distinct policy or programme compared to 13 pairs for Burkina Faso covering five policies, of which three were referenced by at least three programmes, and eight programmes, of which four referenced more than one policy (Figure 4). In Nigeria, these pairs were across the ‘agriculture’ and ‘nutrition’ focus area, while in Burkina Faso, pairs represented the ‘health, ‘social’, ‘agriculture’ and ‘nutrition’ focus areas, showcasing stronger inter-linkages between the different focus areas. None of the linked pairs were assessed to have complete coherence for more than four of the nine external analysis questions, meaning that programmes did not align to the policies they referenced for some or any of the analysis questions representing the process steps in the analysis framework.

We observed variation in the degree of coherence across analysis questions. We found only a few cases (pairs 2, 6, 9 10, 11, 12 and 13 for Burkina Faso and pairs 2 and 3 for Nigeria) where there was complete alignment across a pair for a given process step (i.e. EAQ-4—are policies and programmes identifying similar objectives). For process steps with more than one analysis question [EAQ-1, EAQ-2, EAQ-3 (context) and EAQ-6, EAQ-7, EAQ-8 (interventions)], we find examples of complete coherence for up to two of the three analysis questions (e.g. pairs 1, 3, 6, 8, 9, 10, 11 and 13 for Burkina Faso and pair 3 for Nigeria) but no cases where there was complete coherence for all three of the analysis questions for either of these process steps. Of the policy/programme pairs in Burkina Faso that reference the same policy, we see commonalities in the external coherence assessment. For example, of the four pairs that reference BF16*, the coherence assessment is the same across five of the nine EAQs for all four pairs. Of the policy/programme pairs in Burkina Faso that reference the same programme, we see some with similar assessments of coherence (e.g. pairs that link to programme BF18), but this is not consistent (e.g. pairs that link to programme BF24 do not have a consistent assessment of coherence).

#### Narrative synthesis of external coherence

We found key information missing in both the programme and policy from the pairs for most process steps (context, nutrition indicators, interventions and coverage indicators), which hindered making comparisons and determining the level of alignment between both documents. For example, for the question ‘are policies and programmes targeting the same beneficiary groups/geographical areas?’ under process step 4 (interventions), the following pairs in Burkina Faso (1, 2, 3 and 4) were deemed to have no coherence because the policy documents did not present or only partially presented interventions/planned activities and hence did not mention any beneficiary group. For the question ‘are policies and programmes identifying the same nutrition indicators’ under process step 3 (nutrition indicators), the reasons for no coherence is based on the fact that some pairs did not have common nutrition indicators between the policy and programme documents (pairs 1 and 2 for Nigeria; pairs 3 and 10 for Burkina Faso), while for other pairs (5, 6, 7, 8 and 13 for Burkina Faso), coherence could not be assessed as nutrition indicators were missing from the policy documents. Summary examples by assessment category from policy and programme documents are presented in Table 3 for the external coherence.

**Table 3.** Summary examples from policy and programme documents by assessment category—external coherence

		Level of coherence		
Analysis questions		None	Partial	Complete
1. Context	EAQ-1. Are policies and programmes highlighting similar problems?	The policy cites prevalence of under 5 (U5) stunting, wasting and underweight in addition to widespread deficiency in vitamin A, iron and iodine. The programme context focuses solely on agriculture and poverty without mention of nutritional status. —Pair 1 (N3/N21)	The policy focuses on stunting and acute malnutrition among children U5, micronutrient deficiencies (anaemia, iodine deficiency and other vitamin deficiencies) among U5, pregnant and lactating mothers, nutrition-related non-communicable diseases (NR-NCDs) (obesity and hypertension) and drivers of these forms of malnutrition, including climate change, market/food prices, unhealthy diets and poor diet diversity, poor health and health literacy, WASH (water, sanitation, and hygiene), food insecurity and poor health and nutritional services. The programme focuses primarily on U5 wasting (acute malnutrition), but also mentions U5 stunting (impaired growth) and drivers of undernutrition, such as food insecurity, as a result of weak agricultural systems, demographic growth, price of food products and poverty. **—**Pair 5 (BF1–BF23)	The policy focuses on U5 stunting, acute malnutrition, and vitamin deficiencies (vitamin A, iodine, iron deficiency) among children and its drivers, such as hunger and food insecurity. The programme focuses on U5 stunting and acute malnutrition, underweight among children, anaemia among pregnant women and children and drivers of these forms of malnutrition, such as food insecurity, agricultural production, poverty, gender inequalities, poor diet diversity and food consumption, inadequate maternal and childcare, certain diseases (diarrhoea and malaria), limited access to safe water, sanitation and health services, migration and security challenges, climate change, poor nutrition systems and lack of data, and limited government and funding restrictions. Overall, there is full coherency for the forms of malnutrition and for some drivers. **—**Pair 10 (BF15*–BF24)
	EAQ-2. Is there a match between policies and programmes for populations identified as most affected**?**	The policy focuses on children, whereas the programme focuses on the adult population (males and females) and the elderly. —Pair 3 (BF6*/BF9*)	Both the policy and programme focus on the nutrition status among the U5 population. The programme also identifies nutrition challenges among women of reproductive age (WRA). —Pair 2 (N15–N16*)	Both the policy and programme focus on the nutrition status among children under 5 years of age and women of reproductive age. —Pair 3 (N1–N24)
	EAQ-3. Is there a match between policies and programmes implemented for disparities: – Geographical areas – Other (e.g. socio-economic)	The policy provides some geographical disaggregation, whereas the programme provides contextual information stratified by gender and urban/rural status, but not by geographical areas. There are no socio-economic disparities discusses in either the policy and the programme. **—**Pair 3 (BF6*/BF9*)	BF12*–BF24: The policy document provides some geographical disaggregation (e.g. by regions), whereas the programme presents some geographical, gender and other (seasonality) disaggregation. No urban/rural or socio-economic disparities are discussed in both the policy and the programme documents. **—**Pair 12 (BF12*–BF24)	Both the policy and programme do not specify any geographical, gender or socio-economic disparities. **—**Pair 11 (BF15*–BF18)
2. Objectives	EAQ-4. Are policies and programmes identifying related objectives?	The policy and programme are well aligned in their objectives to enhance agricultural productivity and also consistent with one another in the absence of any nutrition objectives. The programme does include an objective to achieve food security. **—**Pair 1 (N3/N21)	The policy main objectives are around food and nutrition security and include food availability, capacity building for food and nut crises, improving physical and financial access to food, reinforcing food and nutrition security governance and improve food diversity in the households. The programme main objective is to contribute to food security, improved access to safe water and improved sanitation, as well as improved governance and health & health services. Although both, the policy and the programme focus on food security, not all objectives are in alignment. Therefore, the policy and the programme are in partial alignment. **—**Pair 5 (BF1–BF23)	Both the programme and policy seek to reduce malnutrition among women and children under 5. The policy generally seeks to improve food security with the aim to improve a number of specific nutrition outcomes including micronutrient deficiency and under nutrition and prevention of diet-related non-communicable diseases (NCDs). The programme aims to strengthen the Nigerian Nutrition System with improved implementation of the National Multi-Sectoral Strategic Plan of Action for Nutrition, improved funding for nutrition, and increased momentum for the Scaling up Nutrition movement in Nigeria. **—**Pair 3 (N1–N24)
3. Nutrition indicators	EAQ-5. Are policies and programmes identifying the same nutrition indicators? What are the differences between these nutrition indicators in policies and programmes?	The policy identifies U5 stunting as a nutrition indicator. The programme identifies obesity, diabetes, hypertension and salt consumption as nutrition indicators. **—**Pair 3 (BF6*/BF9*)	There are two shared indicators between the policy and programme: (1) reduction of stunted U5 children (both documents cite the same statistic of 36% stunting in 2013. The programme sets a slightly more ambitious target than the policy, but the policy does not specify the timeframe for meeting the target) and (2) appropriate complementary feeding after 6 months (the policy specifies this indicator as Minimum Acceptable Diet (MAD) for 6–23 months, but the programme does not have this level of detail). The programme generally seeks to reduce malnutrition and food security by 50% but does not specify the indicators for measurement. Besides the two indicators shared with the policy, the programme also includes exclusive breastfeeding as an indicator. The policy specifies several other nutrition indicators. The World Health Assembly (WHA) indicators include targets to reduce stunting, wasting, childhood overweight, low birthweight and anaemia among WRA. Beyond these, the policy also includes targets to reduce obesity among WRA, the Global Hunger Index score, Food Consumption Score and proportion of agricultural budget that is spent on nutrition activities. **—**Pair 3 (N1–N24)	No examples
4. Interventions	EAQ-6. Are programmes implementing the interventions outlined in policy documents?	The policy and programme both focus interventions on value addition of agricultural production. The programme includes considerable detail on the intervention approach, but there are no nutrition interventions specified. The policy includes nutrient fortification as value addition. **—**Pair 1 (N3/N21)	The policy document does not propose any interventions. The programme document proposes interventions, including activities that reduce the maternal and infant mortality and activities that promote food security (such as agriculture), access to safe water and improved sanitation, particularly among the most vulnerable populations. **—**Pair 5 (BF1–BF23)	Both the policy and programme seek to use a social mobilization strategy to employ advocacy tools to influence policy-makers. Both also include monitoring and evaluation (M&E) activities. The programme also includes capacity building for effective delivery of high-impact nutrition interventions and partnership and coalition building among key stakeholders. The policy lists several service delivery interventions including behaviour change communication (BCC), delivery of bio-fortified crops, and promotion of high-energy foods. **—**Pair 3 (N1–N24)
	EAQ-7. Are policies and programmes targeting the same beneficiary groups/geographical areas?	The policy does not mention any targeted population groups or geographical areas, given that it does not propose any interventions. The programme document targets primarily the adult population, as well as health professionals, including doctors and nurses. **—**Pair 3 (BF6*/BF9*)	Both the policy and programme identify mothers as the primary target beneficiaries. The policy also includes infants as target beneficiaries. The programme does not name infants but does specify that women who are pregnant, lactating and with children under the age of 2 years are prioritized with the aim to improve child nutrition outcomes. Other target beneficiaries are also mentioned in both the policy and programme, including WRA, fathers, other caregivers, wet nurses, grandmothers/mothers in law, health workers, community volunteers (CVs) and Traditional Birth Attendances (TBAs), community leaders, CBOs, vulnerable groups. —Pair 1 (N15–N16*)	The policy mentions women and children, as well as other groups (overall population, youth/adolescents and marginalized people). The program document targets women, infants (0–2 years old) and vulnerable households with a focus on the population living in rural areas. Hence, there is alignment for the beneficiary groups, as both include children, women and vulnerable populations. **—**Pair 11 (BF15*–BF18)
	EAQ-8. Are policies and programmes involving similar implementers?	There is close alignment between the policy and programme, which both name the Federal Ministry of Agriculture and Rural Development (FMARD) and State Ministries of Agriculture as the lead implementing agency. Both name other key stakeholder groups involved in implementation. The programme specifies the management structure within the MoA and describes how activities will be conducted in coordination or collaboration with other relevant government agencies and service providers such as non-governmental organizations (NGOs), and private consultants/contractors. The policy identifies private investors and development partners as potential financial support and also lists community groups, NGOs, and community-based organizations (CBOs) as other key actors, although does not specify their role. **—**Pair 1 (N3/N21)	The implementers listed in the policy are: government, communities, private sector and NGOs. The implementers mentioned in the programme are: government, private sector and NGOs. There is overlap for government, private sector and NGOs. However, the policy mentions communities, which is not included in the programme. **—**Pair 5 (BF1–BF23)	Both the policy and programme involve government, communities, research/academia, NGOs, private sector and the media as implementers or implementing partners. The programme focuses on actors involved in community-level implementation, specifying community groups and individuals while the policy specifies broader stakeholder categories. —Pair 1 (N15–N16*)
5. Coverage indicators	EAQ-9. If coverage indicators are identified, are they consistent across policies and programmes?	Coverage indicators are only proposed in the programme document. **—**Pair 3 (BF6*/BF9*)	Both the policy and programme documents focus on the coverage of infant and young child feeding practices (IYCF) interventions but differ in the measurement of coverage. The programme specifies coverage in terms of delivery channels (e.g. traditional birth attendants supporting mothers with optimal IYCF practices), while the policy specifies coverage of programmes to target beneficiaries (e.g. mothers encouraged to exclusively breastfeed her infant until 6 months). Both include targets for outcome measures. In addition, both documents set out plans for an M&E system that would likely identify further coverage indicators. —Pair 2 (N15–N16*)	No examples

## Discussion

This study offers a novel approach to understanding the structure of, and interplay between, the policy and programme landscapes for nutrition at the national level in two West African countries, Burkina Faso and Nigeria. The policy coherence concept is widely applied in the literature, which details different analysis approaches, e.g. assessment of political commitment and capacity for nutrition policy in LMICs through desk reviews and qualitative assessments (Chopra *et al.*, 2009; Engesveen *et al.*, 2009; Harris *et al.*, 2017; Poole *et al.*, 2018; Ouedraogo *et al.*, 2020). Yet, few attempt to systematically assess coherence between policies and programmes and determine if planned actions align to the established priorities. This study adds to the existing evidence on nutrition policy coherence by introducing a coherence framework to analyse the content of policies and programme documents.

### Summary of key findings and interpretation

#### Internal coherence

Our analysis revealed that nearly all policy and programme documents from both case study countries had partial internal coherence. Only six documents (12%) across the two countries were assessed to have complete coherence for at least three of the four internal analysis questions. We find that policies and programmes with an explicit nutrition focus generally have a higher level of internal coherence for nutrition. Of the six documents with the highest assessment of internal coherence, four were in the focus area of ‘nutrition’ (three from Nigeria and one from Burkina Faso) and two were policy documents in the ‘health’ focus areas from Burkina Faso. This finding shows that policies and programmes with an explicit nutrition focus generally have a higher level of internal coherence for nutrition, which is consistent with other studies on nutrition policy coherence. A study by Pelletier *et al*. (2012) examines the integration of nutrition in policies across different sectors and identifies barriers to implementation in sectors outside the health sector (e.g. agriculture and education), including fewer resources to support nutrition-related activities and challenges in coordination, planning and decision-making for nutrition. Similarly, Ouedraogo *et al*. (2020) identified variations in the extent to which nutrition is integrated into different sector policies in Burkina Faso. However, our results show that Burkina Faso policy and programme documents outside the nutrition focus have a higher level of internal coherence for nutrition compared to Nigeria documents. A review of studies that examined the enabling environment for improvements in nutrition affirm that actions across multiple sectors are essential for achieving stunting reduction (Heidkamp *et al.*, 2021), which is reflected by studies following this methodology in both Nigeria (Adeyemi *et al.*, 2021) and Burkina Faso (Turowska *et al.*, 2021).

We observed missing process steps, lack of clarity and specificity (e.g. insufficient information in the situation analysis or general/unclear nutrition objectives), and inconsistencies (e.g. a different target population identified across process steps) as the primary reasons for lower coherence on individual analysis questions. In some instances, the proposed interventions or activities only partially addressed the forms of malnutrition described in the situation analysis or objectives of the document. A study on interactions between different environmental policy areas in the European Union also found variations in coherence between objectives, actions and implementation practices (Nilsson *et al.*, 2012). Our findings highlight the need for better alignment on nutrition in document drafting to increase the likelihood that policies and programmes achieve their intended outcomes, which requires a strong policy process for nutrition at all stages (i.e. commitment, agenda-setting, policy formulation and implementation). The literature affirms that real political commitment that includes allocation of necessary resources, accountability, and oversight in policy formulation and implementation is needed to bring nutrition to the agenda and develop operational plans (Pelletier *et al.*, 2012; Fox *et al.*, 2015). Research in South Africa finds that beliefs and paradigms of policy actors can create barriers to achieving coherence that benefits nutrition (Thow *et al.*, 2018). Other factors that may hinder political commitment and agenda-setting for nutrition encompass lack of awareness of effective interventions, low investments, competing interests across stakeholders, short terms of politicians, and gaps in communication and coordination (Engesveen *et al.*, 2009; Pelletier *et al.*, 2012; Dufour and Dodé, 2016; Hawkes, 2016).

We found that, while some policy documents outline nutrition activities, only seven (14%) include indicators to measure intervention coverage (although a few did mention that indicators would be detailed in forthcoming programme-oriented documents). The process steps for interventions and coverage indicators were also missing (partially or completely) from several of the programme documents. Evidence on intervention coverage is noted as a key component to support a strong enabling environment for nutrition in Nigeria (Adeyemi *et al.*, 2021). Furthermore, Avula *et al*. (2021) identified that one of the factors contributing to reduced stunting in four Indian states was improved coverage of health and nutrition interventions. Failure to include monitoring and evaluation plans into policy or programme documents negatively impacts the scaling-up process and reduces governance accountability (Gillespie *et al.*, 2015). Ruel and Alderman (2013) also highlight the need for nutrition-sensitive programmes to improve targeting and strengthen their nutrition objectives and actions. When nutrition-sensitive programmes are purposively designed with clear target groups for the nutrition objectives, standardized nutrition outcomes and rigorous evaluation designs, they have been shown to be more effective along various nutrition outcomes (Ruel *et al.*, 2018).

#### External coherence

We found few linkages between policy and programme documents, whereby a programme explicitly references and responds to a policy, although there is a contrast between the case study countries with only three policy/programme pairs identified for Nigeria compared to 13 pairs for Burkina Faso. We see considerable potential to improve programme responsiveness to nutrition-relevant policies in Nigeria considering that 19 policies were identified across a range of focus areas, but there are only eight programmes at the national level to implement these policies. It is, however, important to note that any policy must be ratified at the state level before it can be operationalized in a programme, which may contribute to the low level of external coherence observed in Nigeria. A review of five national food- and climate-related policies in Nigeria examined coherence for diet and climate and concluded that Nigeria lacks a coherent policy environment to fully support food and climate actions (Morgan and Fanzo, 2020). In Burkina Faso, the Multisectoral Nutrition Strategic Plan has aided in successful coordination on nutrition policies and programmes across sectors, which likely contributes to the greater number of policy and programme links observed in comparison to Nigeria. However, even with this coordinating structure in Burkina Faso, we see a lack of coherence at the process step level across the linked pairs, suggesting that policy priorities are still not fully realized in programmes. A study examining factors that have led to the reduction in stunting in Burkina Faso also pointed to existing coordination for nutrition across sectors but notes that more can be done to coordinate at a decentralized level (Turowska *et al.*, 2021). Such gaps between policy priorities and planned actions are seen in other contexts; for example, Harriss and Kohli (2009) describe how the political landscape at the state level in India affects the capacity for nutrition programme responses.

Our study purposively analysed explicit pairs as a proxy to examine coherence across policies and programmes. Other studies propose possible explanations for low inter-linkages including (1) insufficient levels of communication and coordination between policy and programme planners; (2) development agencies and donors driving programme content/programmatic actions according to external priorities rather than aligning with national nutrition priorities as depicted in their policies (Van Royen *et al.*, 2013); (3) tensions between the policy and programme objectives within and between sectors (Ruel *et al.*, 2018; Thow *et al.*, 2018); (4) lack of capacity at different levels (individual, organizational and systemic) (Gillespie *et al.*, 2013) and (5) policy-makers and implementers having different understandings of nutrition problems and solutions in the country. Thus, strengthening capacity for cross-sectoral coordination and improving governance of policy-programme making processes are two strategies that can help achieve alignment of policies and programmes across sectors (Hawkes, 2016).

Furthermore, we find that among the linked policy/programme pairs, no programme was fully aligned to the referenced policy. Gaps in internal coherence for a given document (e.g. information missing in the document for a process step) reduces the potential for strong external coherence. Other reasons for low levels of external coherence included pairs lacking common nutrition indicators or nutrition indicators inconsistently defined. These findings raise the question whether policies fully reflect the current nutrition priorities, which could be a result of a dynamic nutrition situation (e.g. rapid increase of overweight and obesity, emergency situations) in relation to a longer policy cycle. Ruel and Alderman (2013) emphasize the need for policies and programmes to support nutrition outcomes across sectors. Ideally, policies from different sectors create a coherent push for nutrition (as exemplified in Burkina Faso), which then translates to programmes. There are many complexities to actualizing this process: supportive policies in one sector can be made irrelevant by policies or practices in others (Hawkes, 2016); involving many different sectors with their own conflicting and competing political agendas and objectives limits how much can be done to include nutrition; and integrating multiple interventions from different sectors into programmes increases the risk of difficulties to implement and scale up with quality (Ruel and Alderman, 2013). Not all policies and programmes should include nutrition, nor is it always desirable due to potential competing sectoral interests. Other studies have shown that a sectoral policy can be effective in generating an impact on, for example, stunting reduction, when it achieves its key objectives without including nutrition (Heidkamp *et al.*, 2021). Yet, if a policy or programme has a nutrition objective, it should have activities, outcomes and indicators that permit the tracking of its impact with regard to these objectives. Ensuring dialogue across sectors at the planning, monitoring and review stages of policy while making sure each sector implements the outlined programmes with quality and efficiency is essential (The World Bank, 2013). A more pragmatic approach could be co-location of sectoral programmes rather than coordinating the development and delivery of fully integrated programmes (Ruel *et al.*, 2018; Heidkamp *et al.*, 2021).

### Study strengths and limitations

The evidence in this paper relies on a robust, comprehensive and systematic assessment of policy and programme coherence for nutrition at the country level. This novel approach comes with the following strengths and limitations. This study offers a new approach for the assessment of policy and programme coherence for nutrition, which is an identified gap in methods (Parsons and Hawkes, 2019). Rather than focusing solely on the assessment of coherence of the policy landscape, we introduce an analytical approach to assess internal coherence within documents and the translation from policies to programmes. This approach may be replicated in different country contexts and for issues outside of nutrition. For example, food systems have been receiving increased attention recently, and improved policy coherence has been proposed to address food system challenges (Parsons and Hawkes, 2018). Understanding gaps in coherence can help to strengthen policy and programme design in the planning stage (Huttunen *et al.*, 2014). It is also important to note limitations to this study and the proposed approach. The intensive nature of this approach may limit the practicality of replication for assessments in other contexts. The assessment of policies and programmes was limited by document availability at the time of the searches (e.g. draft versions). To mitigate the risk of missing important closed source documents, the complementary search approach included targeted website searches, Google searches for official documents and grey literature, and consultation with key national stakeholders in the nutrition community. This study used a documentary and framework analysis approach to assess coherence, which does not allow us to capture the complexities between the policy and programme nexus from the viewpoint of actors and is limited by data availability, and the type of document available for review. Also to note, this study assesses the coherence of policies and programmes using an impact pathway approach yet does not attempt to assess the impacts of any policies or programmes on nutrition outcomes. We focus on the consistency and alignment within and across policy and programme documents as a means to improve their impact on nutrition outcomes.

## Conclusion

Our findings, across countries, showed that internal coherence remains low outside of the nutrition and health sector and reinforces the importance of coherence for nutrition within different sectors. We also found that there is a limited degree of alignment between policies and programmes. There is ample opportunity to increase both internal and external coherence for nutrition within nutrition-relevant policies and programmes to support a unified nutrition agenda.

## Supplementary Material

czab108_Supp

## Data Availability

The data underlying this article will be shared on reasonable request to the corresponding author.
