# Supplementary material for: Coherence for nutrition: insights from nutrition-relevant policies and programmes in Burkina Faso and Nigeria
Source: Health Policy Plan. 2021 Sep 21;36(10):1574–92. doi: 10.1093/heapol/czab108 (PMC8597973; doi:10.1093/heapol/czab108)
Supplement: czab108_Supp [file czab108_supp.zip › Nutrition policy coherence_insights from West Africa_supplementary materials.docx]

**Supplementary Material 1:** Search strategy

| **Search approach** | **Policies** | **Programmes** |
| --- | --- | --- |
| **Targeted website search** | Specific websites (online databases and resources) were searched to find relevant policies for Nigeria and Burkina Faso. These included, but were not limited to: government ministries, development agencies (e.g. FAO, UNICEF, WHO), (I)NGOs (e.g. ACF, Save the Children), regional agencies websites (WAHO, ECOWAS, WANCDI) | Specific websites (online databases and resources) were searched to find relevant programmes for Nigeria and Burkina Faso. These included, but were not limited to: government ministries, development agencies (e.g. FAO, UNICEF, WHO), (I)NGOs (e.g. ACF, Save the Children), regional agencies websites (WAHO, ECOWAS, WANCDI), Research institutes (IFPRI) |
| **Google search** | We searched Google for policy documents in each country using the following search strings:  [Nigeria] AND [policy OR policies OR “action plan” OR strategy] AND [nutrition].  [Burkina Faso] AND [policy OR policies OR “action plan” OR strategy OR politique* OR “plan d’action” OR stratégie] AND [nutrition].  The first 100 hits were screened for relevance and included if they met the inclusion criteria. | We searched Google for programme documents in each country using the following search strings:  [Nigeria] AND [program OR programme OR project OR plan OR strategy OR intervention OR initiative] AND [nutrition].  [Burkina Faso] AND [program OR programme OR project OR projet OR plan OR strategy OR stratégie OR intervention OR initiative] AND [nutrition].  [name of country] AND [program OR programme OR project OR projet OR plan OR strategy OR stratégie OR intervention OR initiative] AND [nutrition] site: websites (e.g. scalingupnutritition.org)  The first 100 hits were screened for relevance and included if they met the inclusion criteria. |
| **Consultation with in-country content experts via email** | Targeted consultations with regional and in-country experts were used to access documents difficult to obtain, working documents (e.g. drafts of policies) or otherwise non-public items. Experts included people from government, UN, NGOs and Civil Society, DataDENT, WAHO, partners of the Stories of Change project. | Targeted consultations with regional and in-country experts were used to access documents difficult to obtain, working programme documents (e.g. drafts of policies) or otherwise non-public items. Experts included people from government, UN, NGOs and Civil Society, DataDENT, WAHO, partners of the Stories of Change project, A&T. |
| **Reference search** |  | Policies were reviewed to see if they refer to any programmes which were not included through the above search. |

**Supplementary Material 2:** Coding tree, coding and analysis questions for policy and programme review

| **Process step** | **Policies** | | **Programmes** | | **External coherence** |
| --- | --- | --- | --- | --- | --- |
|  | **Coding nodes/sub-nodes** | **Coding Question(s)** | **Coding nodes/sub-nodes** | **Coding Question(s)** |  |
| **1. Context** | Assess context/nutrition situation | **What context and**  **problems do the policies**  **highlight and focus on?**   - What nutrition challenges are identified in policy documents? (Underw/overw/micronutrient deficiency). Any WHA targets? - Disparities? (Urban/rural, National/sub-national, Income level, Gender) - What populations are most affected? - What are the main drivers? - Is it multi-sectoral? - Is the nutrition context evidence-based? | Nutrition situation   - Malnutrition forms   - WHA targets   - Other - Drivers:   - WHA targets   - Other - Consequences - Populations - Type of populations affected - Geographical areas affected - Disparities - Gender - Geographical - Urban/rural - Other - Evidence-based (i.e. whether figures/evidence provided to support the situation analysis) - Evidence base for drivers/consequences (with & without references) - Evidence base for intervention choice/development (with & without references)   Note: When not coded, this means no referencing. | **What context and problems do the programmes highlight and focus on?**   - What forms of malnutrition exist? (Underw/overw/micronutrient deficiency). Any WHA targets? - What are the main drivers of the current nutrition situation? - What are the consequences of the current nutrition situation? - What populations are most affected? - Disparities? (Urban/rural, National/sub-national, Income level, Gender) - Is the nutrition context evidence-based? | Are policies and programmes highlighting similar problems?  Is there a match between policies and programmes for disparities:   - Geographical areas - Other (e.g. socio-economic) |
|  | [Full list of policies – see final policy brief]  OR  Intervention/programme | - Are programmes referenced in policy documents? What does this policy say about existing/planned/proposed interventions, programmes, projects, commitments? | Related policy & laws   - Past policies - Current policies | - Is the programme framed in the context of an existing policy (from policy landscape)? |  |
|  |  |  | Related programmes   - Past programmes - Current programmes | - Are any other programmes referenced? Past programmes / Current programmes  *Note: past programmes will be useful as part of the full picture on context/ evidence-based* |  |
| **2. Objectives** | General objectives   - Nutrition general objectives | - What are the general objectives/visions of the policies? - What nutrition objectives are identified?   Within the General Objective of this policy, is there a nutrition (-specific or -sensitive) objective? | General objectives | - What are the general objectives of the programme? | Are policies and programmes identifying related objectives? |
|  | Specific objectives   - Nutrition Specific Objectives | What are the Nutrition Specific Objectives of this policy? | Nutrition objectives | - What nutrition objectives are identified (i.e. general and/or specific)? |  |
|  | Targeted beneficiaries   - Nutrition_Young children - Nutrition_Women of reproductive age - Nutrition_Adults | What beneficiaries are targeted by this policy? |  |  |  |
|  | Actors   - multisectoral coordination | Who plays a role in these policies? What role(s)? Are there challenges highlighted (if so, what?)?  What are the coordination mechanisms described to coordinate actors across different sectors? Are coordination mechanisms described up to decentralised level to ensure that it is multisectorial? | Coordination | Who plays a role in these programmes? What role(s)?  What are the coordination mechanisms? |  |
| **3. Nutrition indicators** | Nutrition indicators | Are there *nutrition* indicators listed in this policy to measure success/implementation?   - Identify which of the 17 key nutrition indicators from Phase I are specified to track progress towards the stated objectives. - Are there targets for indicators? If so, what are the target values and start and end dates? - What kinds of indicators are there (input, output, outcome…)? - Are they dissagegated? | Key nutrition indicators & WHA targets | - Are any of the 17 key nutrition indicators/WHA targets identified/listed in the document?  - What kinds of indicators are there (input, output, outcome…)?  - Are they disaggregated? | Are policies and programmes identifying the same nutrition indicators? |
|  | Nutrition indicators | - Which other nutrition indicators are specified to track progress towards the stated objectives? | Other nutrition indicators | - Are any other nutrition indicators (besides the 17/WHA targets) identified in the document?  - What kinds of indicators are there (input, output, outcome…)?  - Are they disaggregated? |  |
|  | Nutrition indicators  Monitoring Evaluation & Accountability? | - Is a data source specified for the nutrition indicator(s)? | Nutrition indicators – data sources:   - Key nutrition indicators - Other nutrition indicators | - Is a data source specified for the nutrition indicator(s)? |  |
| **4.Interventions** | Planned nutrition activities listed  Intervention/programme  Targeting of beneficiaries   - Nutrition_Young children - Nutrition_Women of reproductive age - Nutrition_Adults | What Gillespie et al. 24intervention/programme are referred to in the policies?  Which beneficiaries are being targeted by the policy for these Gillespie et al. 24 intervention/programmes? | Intervention type   - Gillespie et al. 24 interventions - Other interventions   Intervention objective   - Gillespie et al. 24 interventions - Other interventions   Geographical coverage   - Gillespie et al. 24 interventions - Other interventions   Intervention beneficiaries   - Gillespie et al. 24 interventions - Infants under 5 yrs - Child 0-6 months - Child 6-23 months - Child 6-59 months - Other combination - WRA 15-49 yrs - Other interventions - Infants under 5 yrs - Child 0-6 months - Child 6-23 months - Child 6-59 months - Other - Children aged 5-9 yrs - Adolescents aged 10-14 yrs - WRA 15-49 yrs - Mixed age group - Other   Intervention evidence   - Gillespie et al. 24 interventions - Other interventions   Intervention implementation (activities)   - Gillespie et al. 24 interventions - Other interventions   Intervention implementers   - Gillespie et al. 24 interventions - Other interventions   Intervention financing   - Gillespie interventions - Funding - Costing - Other interventions - Funding - Costing   Intervention coordination – actors and roles of actors   - Gillespie et al. 24 interventions - Government (national or local) - Communities - Private sector - Donors - Research & academia - UN agencies - Other interventions - Government (national or local) - Communities - Private sector - Donors - Research & academia - UN agencies | - Does the document propose [any of the 24 intervention] listed in Gillespie et al. 2019*?* - Does the document propose any other intervention (than Gillespie et al. 24)? - *Intervention type* (what interventions/activities are proposed with an intended nutrition outcome) - *What is the objective of the intervention? Programme?* - *Geographical coverage* - *Intervention beneficiaries* (what is the target population of the intervention?) - *Intervention evidence* (does the document cite evidence to support the effectiveness of the intervention/activity?) - *What are the activities proposed/implemented?* - *Intervention implementers* (what institution is responsible for implementing the interventions?) - *Intervention funders* (what funders are responsible for implementing the interventions?) – Is there a costing for the proposed interventions? - *What are the coordination mechanisms of the intervention(s) proposed?* | - Are programmes implementing the interventions outlined in policy documents? - Are policies and programmes targeting the same beneficiary groups/geographical areas? - Are policies and programmes involving similar implementers?   *[note: cross-reference with node on actors under objectives process step]* |
|  | Planned nutrition activities listed  Intervention/programme  Targeting of beneficiaries  Nutrition_Young children  Nutrition_Women of reproductive age  Nutrition_Adults | - Which other interventions are proposed in any policy document?  - Which beneficiaries are being targeted by the policy in other interventions? |  |  |  |
| **5.Coverage indicators** | Nutrition indicators  Monitoring Evaluation & Accountability? | Are any indicators specified to measure the coverage of the intervention (whether or not a target is specified)? | Indicators   - Gillespie et al. 24 interventions - Other interventions | - Are coverage indicators specified?  If so which ones? | If coverage indicators are identified, are they consistent across policies and programmes? |
|  | Nutrition indicators  Monitoring Evaluation & Accountability? | Is a data source specified for measuring coverage and does the data source include such an indicator? | Data sources   - Gillespie et al. 24 interventions - Other interventions | - Is a data source specified for measuring coverage? |  |
| **Internal coherence** |  | What is the internal coherence at the individual policy level?   - Are policy objectives aligned with the identified challenges? - What is included in the policies to address the highlighted problems? - Is there coherence between context, nutrition objectives, nutrition indicators, planned nutrition activities, and budget for nutrition? (e.g. if there’s a specific objective on stunting, is there an indicator on stunting?)? |  | What is the internal coherence at the individual programme level?   - Which challenges are well addressed by proposed interventions, and which are not well addressed? - Are the interventions based on existing evidence to effectively address the challenges or meet the objectives? - Does the document cite the evidence? - Is there coherence between context, objectives, indicators, and interventions?   - Are programme objectives aligned with the identified challenges and drivers?   - Do the identified indicators align with the stated objective?   - Does the programme include interventions to address the identified challenges and drivers?   - Does the identified coverage indicator(s) appropriately measure coverage of the specified intervention(s)?   - Overall assessment of individual programme coherency based on the questions above |  |
|  |  | **Summary of internal coherence across policies?**  **Key descriptive questions**   - Nr of policies that provide context? - How many policies cover any of the 17 nutrition indicators? Cover the WHA targets? - Which indicators are covered most frequently, and which indicators are most infrequent or completely excluded from any policy documents?   **Are there differences across attributes? For key nodes?** |  | **Summary of internal coherence across programmes?**  **Key descriptive questions**   - Nr of programmes that provide context? - How many programmes cover any of the 17 nutrition indicators? Cover the WHA targets? - Which indicators are covered most frequently, and which indicators are most infrequent or completely excluded from any programme documents? - How many programmes cover any other nutrition indicators? - Which indicators are covered most frequently, and which indicators are most infrequent or completely excluded from any programme documents?   **Are there differences across attributes? For key nodes?** |  |
